# Analysis of a case series of behavioral variant frontotemporal dementia: emphasis on diagnostic delay

**DOI:** 10.1590/S1980-57642013DN70100009

**Published:** 2013

**Authors:** Henrique Cerqueira Guimarães, Thiago Cardoso Vale, Victor Pimentel, Nayara Carvalho de Sá, Rogério Gomes Beato, Paulo Caramelli

**Affiliations:** 1 Cognitive and Behavioral Neurology Unit, Neurology Service, Hospital das Clínicas of the Federal University of Minas Gerais, Belo Horizonte MG, Brazil.; 2 Department of Internal Medicine, Faculty of Medicine, Federal University of Minas Gerais, Belo Horizonte MG, Brazil.; 3 Cognitive and Behavioral Neurology Unit, Neurology Service, Hospital das Clínicas of the Federal University of Minas Gerais, Belo Horizonte MG, Brazil. Department of Internal Medicine, Faculty of Medicine, Federal University of Minas Gerais, Belo Horizonte MG, Brazil.

**Keywords:** frontotemporal dementia, Alzheimer's disease, dementia, diagnosis

## Abstract

**INTRODUCTION:**

Despite many advances in the characterization of the behavioral variant of frontotemporal dementia (bvFTD), the diagnosis of this syndrome poses a significant challenge, while delays or diagnostic mistakes may impact the proper clinical management of these patients.

**OBJECTIVE:**

To describe the clinical profile at first evaluation of a sample of patients with bvFTD from a specialized outpatient neurological unit, with emphasis on the analysis of the delay between the onset of symptoms and diagnosis.

**METHODS:**

We selected 31 patients that fulfilled international consensus criteria for possible or probable bvFTD. Patients' medical admission sheets were thoroughly reviewed.

**RESULTS:**

Patients' mean age was 67.9±8.2 years; 16 (51.6%) were men. Mean number of years of formal education was 7.7±4.0 years. Mean age at onset was 62.2±7.7 years, indicating a mean of 5.8 years of diagnostic delay. Thirteen patients (41.9%) presented with initial behavioral complaints only, eleven patients (35.5%) had mixed behavioral and memory complaints, five patients (16.1%) presented with memory complaints only, and two patient (6.4%) had behavioral and speech problems. Nine patients (29%) were admitted with alternative diagnoses. Mean and standard deviation scores for the mini-mental state examination, animal category fluency and memory test for drawings (five-minute delayed recall) were 19.3±6.3, 8.3±4.1and 3.7±2.7, respectively.

**CONCLUSION:**

Most patients from this sample were evaluated almost six years after the onset of symptoms and performed poorly on both cognitive screening tests and functional evaluation measures.

## Abstract (pt)

**INTRODUÇÃO:**

Apesar dos avanços na caracterização da variante comportamental da demência frontotemporal (vcDFT), o diagnóstico da síndrome apresenta-se desafiador e atrasos ou erros diagnósticos podem prejudicar o tratamento adequado aos pacientes.

**OBJETIVO:**

Descrever o perfil clínico à primeira avaliação de pacientes com vcDFT de uma unidade neurológica ambulatorial especializada, com ênfase na análise do atraso entre o início dos sintomas e diagnóstico.

**MÉTODOS:**

Selecionamos 31 pacientes que preencheram os critérios internacionais para vcDFT possível ou provável. As fichas de admissão foram minuciosamente revisadas.

**RESULTADOS:**

A média de idade dos pacientes foi 67,9±8,2 anos; 16 (51,6%) eram homens. A média de anos de escolaridade foi de 7,7±4,0 anos. A média de idade de início dos sintomas foi 62,2±7,7 anos, indicando um atraso diagnóstico médio de 5,8 anos. Treze pacientes (41,9%) apresentaram-se com apenas queixas comportamentais, onze pacientes (35,5%) tinham queixas amnésticas e comportamentais, cinco pacientes (16,1%) apresentaram-se apenas com queixas de perda de memória, e dois pacientes (6,4%) com problemas de fala e comportamentais. Nove pacientes (29%) foram admitidos com diagnósticos alternativos. A média e desvio-padrão dos escores do mini-exame do estado mental, fluência verbal de animais e teste de memória de figuras (evocação em cinco minutos) foram 19,3±6,3, 8,3±4,1 e 3,7±2,7, respectivamente.

**CONCLUSÃO:**

A maioria dos pacientes da amostra foi avaliada após uma média de quase seis anos de início dos sintomas e apresentaram baixo desempenho nos testes cognitivos e nas medidas de avaliação funcional.

## INTRODUCTION

The behavioral variant of frontotemporal dementia (bvFTD) is the most frequent presenting phenotype from a wide spectrum of neurodegenerative diseases collectively named Frontotemporal Lobar Degeneration (FTLD). This miscellaneous entity also includes the language-impaired subtypes, Progressive Non-fluent Aphasia (PNFA) and Semantic Dementia (SD); extrapyramidal-predominant clinical phenotypes such as Progressive Supranuclear Palsy (PSP) and Corticobasal Syndrome (CBS); and finally, the bvFTD associated with motor neuron disease.^1^ It remains intriguing as to how a handful of implicated genes can determine two major groups of pathological landmarks i.e. tau pathology and ubiquitin inclusions, albeit so many heterogeneous and overlapping phenotypes.^2^ Indeed, even among subjects with the same genetic mutation, the clinical picture can differ dramatically.^3^

The bvFTD seems to equally affect both sexes, although some series have reported a male predominance.^4^ The mean age of onset is in the sixth decade and a positive family history is found in up to 40% of cases. The degenerative process targets preferentially cortical regions that involve the anterior cingulate, insular and orbitofrontal cortex, and anterior temporal pole, which are essential to value-guided decision-making, motivation, social cognition, emotion identification, emphatic concern, and appropriate personal conduct, with relative preservation of episodic memory.^5^ At disease onset, patients may manifest psychiatric symptoms such as maniform states, compulsive behavior, apathy and even psychotic features^6^ that might be misconstrued as depression or a primary psychiatric disorder, delaying the proper evaluation and clinical management. Moreover, there is a growing body of evidence^7^ that a significant proportion of bvFTD patients can present with severe amnestic symptoms, exhibiting a performance on classical neuropsychological tests that is indistinguishable from that observed in Alzheimer's disease (AD).^8,9^ In contrast, pathologically-confirmed AD patients can be misdiagnosed as bvFTD if behavioral presentation is more salient at onset.^10^

Despite major advances in the characterization of bvFTD,^1^ the diagnosis of this syndrome remains a challenge, and delays or diagnostic mistakes may occur in many patients. These difficulties may be even greater in developing countries where several resources, such as functional neuroimaging, e.g., positron emission tomography (PET) or single photon emission tomography (SPECT) scans, genetic testing, and trained professionals are still scarce.

To our knowledge, scant studies have discussed the issue of diagnostic delay in bvFTD.^11-13^ Nonetheless, it was the Brazilian study^13^ that reported the longest time delay between disease onset and proper diagnosis. However, all of these reports included PNFA and SD patients, in whom the salient language impairment might have facilitated early referral for specialized assessment. In this regard, the primary aim of this study was to provide a summarized clinical picture from a sample of bvFTD patients upon first arrival for evaluation at a cognitive and behavioral neurology outpatient unit, and also to investigate the possible variables associated with delay in specialized assessment.

## METHODS

Thirty-one patients were consecutively selected after medical chart review. Subjects that, on follow-up, fulfilled international consensus criteria for probable or possible bvFTD had their medical admission sheets thoroughly reviewed. Only patients that had their bvFTD diagnosis first ascertained at our unit were included. The study was approved by the Ethics Committee of the Federal University of Minas Gerais, in Belo Horizonte, Brazil.

We collected data on major demographic and clinical features, along with basic neuroimaging data. Salient clinical symptoms at disease onset were obtained through caregiver reports. The first behavioral core diagnostic disorder was used to define the age at onset. All patients were evaluated with a brief cognitive screening battery consisting of the mini-mental state examination (MMSE), semantic category fluency for animals, and a picture drawing episodic memory test.^14^ They were also evaluated by means of the Functional Activities Questionnaire (FAQ)^15^ and Katz's Index of independence in activities of daily living.^16^

According to consensus diagnostic criteria^17^, delayed evaluation was defined as those participants in whom it took more than three years from symptoms onset to diagnosis. Statistics consisted of a descriptive analysis (means±standard deviation), emphasizing the demographic and clinical profile of these patients at first evaluation. We also estimated potential variables that might have been associated with delayed admission, by performing a stepwise logistic regression analysis. Medcalc version 11.6 was used for statistical analysis.

## RESULTS

Table 1 shows the main demographic and clinical data extracted from the admission sheets of the participants. The sample consisted of 31 patients, with a mean age of 67.9±8.2 years at the time of evaluation in our specialized unit. Subjects had 7.7±4.0mean years of formal education, and 16 (51.6%) were men. Patients' clinical history suggested that the onset of disease was on average at 62.2±7.7mean years of age. This data revealed a mean delay of 5.8±3.8 years until evaluation at our unit.

**Table 1:** Main demographic and clinical features along with evaluation performance of the sample.

**Variables**	Age at onset (mean±SD ; range)	62.2±8.2 ; 43-78	
	Age at first evaluation (mean±SD ; range)	67.9±7.7 ; 47-81	
	Delay since onset, years (mean±SD ; range)	5.8±3.8 ; 1-16	
	Years of formal education (mean±SD ; range)	7.7±4.0 ; 0-18	
	Gender (male/female)	16/15	
	Diagnosis after first evaluation (%):		
	bvFTD	70.9%	
	Other	29.1%	
	Diagnostic category after follow-up (n):		
	probable bvFTD	22	
	possible bvFTD*:	9	
	absent progression**	2	
	unspecified neuroimaging features***	7	
	Clinical features at onset (%)		
	behavioral disorder	41.9%	
	memory complaints and behavioral disorder	35.5%	
	memory complaints	16.1%	
	behavioral and speech disorder	6.4%	
		**Observed**	**Expected**
**Evaluations**	Brief Cognitive Screening Battery (mean±SD)		
	MMSE (0-30),	19.3±6.3	>24^†^
	Category fluency (animals/min.)	8.3±4.1	>12^†^
	Memory test for drawings		
	Learning (0-30)	11.1±7.2	>20^†^
	5-minute delayed recall (0-10)	3.7±2.7	>5^†^
	Functional measures (mean±SD)		
	Katz Index of independence in BADL (0-6)	5.1±1.8	6
	Functional Activities Questionnaire (30-0)	16.5±8.8	<5

* Six patients had only structural neuroimaging data.

** both patients also have unspecified neuroimaging features.

*** including abnormal SPECT findings in three patients. BADL: Basic Activities of Daily Living; Numbers between brackets mean range of possible scores from the worst to best performance.

† Weighted average according to 5 levels of schooling ( 0, 1-3, 4-7, 8-11, >11 years).

Almost 30% of the participants had a diagnosis other than bvFTD at the first round of evaluations in the unit, meeting consensus diagnostic criteria only on follow-up visits: four had unspecified psychiatric diagnosis and five supposedly had AD. Regarding clinical presentation at onset, 13 patients (41.9%) displayed only behavioral disorders, 11 (35.5%) had mixed behavioral disorders and memory complaints, five (16.1%) had only memory complaints, and two (6.4%) participants presented with behavioral disorder and motor speech problems without aphasia (both developed features consistent with PSP-spectrum on follow-up).

There were nine participants that did not fulfill probable bvFTD consensus criteria: two because of absent progression, one of whom had a strong family history of bvFTD whereas the other, a conspicuous disinhibited non-amnestic patient, had abnormal SPECT findings with diffuse and heterogeneous perfusion deficits, especially in posterior parietal regions. The other seven participants with possible bvFTD were progressors and did not meet consensus criteria because of unspecific neuroimaging data. All of these cases had mostly diffuse brain atrophy without a typical frontotemporal pattern. Unfortunately, six of these patients had just structural neuroimaging findings, precluding additional conclusions that might have been achieved with PET or SPECT scans.

Data given in Table 1 show that, at first evaluation in the unit, average performance of the participants was unequivocally impaired on all the brief cognitive screening tests, including the episodic memory test. The lower right-hand side column shows the expected scores according to control-reference performance,^18^ weight-adjusted for five levels of formal education. Functional impairment was also salient, with moderate impairment in instrumental activities of daily living (FAQ: 16.5±8.8 points), and mild dependence in self-care (Katz Index: 5.1±1.8points).

Using a stepwise logistic regression model, we tested which clinical variables might have contributed to the delay (>3 years) from onset of symptoms to first evaluation at our specialized unit. The best model (AUC=0.734; 95% CI: 0.545-0.876), considering gender, schooling and age at onset, showed only memory complaints at presentation as a significant variable associated (OR: 0.12; 95% CI: 0.020-0.714) with delayed evaluation.

## DISCUSSION

Most patients in this case series were evaluated in a specialized unit after almost six years from symptom onset and performed poorly on both cognitive screening tests and functional evaluation measures. Even in this specialized unit, almost 30% of the reported patients did not receive accurate diagnosis during the first round of evaluations. In these cases, bvFTD was only ascertained at follow-up visits. Regarding the characteristics of bvFTD, memory complaints were present in almost half of the sample, isolated or associated with behavioral disorders. The presence of memory issues, according to caregiver reports, was a strong predictor of an earlier assessment at the unit.

Previous studies from developed countries that sought to evaluate early manifestations of bvFTD found, on average, around two years of delay from onset to proper diagnosis.^11,12^ In a Spanish case series of 42 mixed FTLD patients, the reported delay was 3.5 years on average. According to Bahia et al.,^13^ in a cohort of FTLD patients from Brazil, almost 75% of the sample received an AD or psychiatric diagnosis prior to a specialized evaluation, and the delay from onset to proper diagnosis was around four years.

It seems quite reasonable that the bvFTD heterogeneous genetic and pathological background potentially gives rise to very complex and variable clinical presentations. In turn, these issues inevitably increase the effort for an accurate and early diagnosis of this syndrome, especially when technological resources are not available. It was only recently, after the new proposed diagnostic criteria,^17^ that most of the research groups became more comfortable to define a bvFTD diagnosis in patients with an amnestic presentation. The finding that memory complaints might have contributed to an early assessment at our unit suggests that the appropriate characterization of psychiatric symptoms, which dominate the clinical picture in the early stages of the disease, may still be a major obstacle to correct identification and treatment of these patients.^19^

Regarding limitations, we should emphasize that the small sample size and the study's retrospective design preclude any generalizations of our findings, although the results were in accordance with a previous report in Brazil. We chose to include nine patients that only met possible bvFTD consensus criteria. These subjects had no better explanation for their clinical presentation. Additionally, except for one subject who had a strong family history of FTLD, these patients did not match the current definition of FTD phenocopy, since they had either progressive deterioration or abnormal neuroimaging findings, even if non-specific. A common limitation of research based on clinical settings is the lack of autopsy-confirmed diagnosis. This caveat could have been reduced in our series by using pathophysiological biomarkers either in cerebrospinal fluid (CSF) or position emission tomography.^20^ It has been previously demonstrated that CSF markers help discriminate bvFTD from AD with high sensitivity and specificity.^21^ The selection of patients according to their CSF biomarkers profile would increase the specificity of our clinical diagnosis,^10^ avoiding the inclusion of AD patients with frontal presentation.
